# Reproducibility of semiautomated body composition segmentation of abdominal computed tomography: a multiobserver study

**DOI:** 10.1186/s41747-019-0122-5

**Published:** 2019-10-30

**Authors:** Lisa Jannicke Kjønigsen, Magnus Harneshaug, Ann-Monica Fløtten, Lena Korsmo Karterud, Kent Petterson, Grethe Skjolde, Heidi B. Eggesbø, Harald Weedon-Fekjær, Hege Berg Henriksen, Peter M. Lauritzen

**Affiliations:** 1 Division of Radiology and Nuclear Medicine, Oslo University Hospital, Oslo, Norway; 2 The Centre for Old Age Psychiatry Research, Innlandet Hospital Trust, Ottestad, Norway; 3 Institute of Clinical Medicine, Faculty of Medicine, University of Oslo, Oslo, Norway; 4 Oslo Centre for Biostatistics and Epidemiology, Research Support Services, Oslo University Hospital, Oslo, Norway; 5 Division of Clinical Nutrition, Faculty of Medicine, University of Oslo, Oslo, Norway

**Keywords:** Body composition, Abdominal fat, Skeletal muscle, Tomography (X-ray computed), Observer variation

## Abstract

**Background:**

Segmentation of computed tomography (CT) images provides quantitative data on body tissue composition, which may greatly impact the development and progression of diseases such as type 2 diabetes mellitus and cancer. We aimed to evaluate the inter- and intraobserver variation of semiautomated segmentation, to assess whether multiple observers may interchangeably perform this task.

**Methods:**

Anonymised, unenhanced, single mid-abdominal CT images were acquired from 132 subjects from two previous studies. Semiautomated segmentation was performed using a proprietary software package. Abdominal muscle compartment (AMC), inter- and intramuscular adipose tissue (IMAT), visceral adipose tissue (VAT) and subcutaneous adipose tissue (SAT) were identified according to pre-established attenuation ranges. The segmentation was performed by four observers: an oncology resident with extensive training and three radiographers with a 2-week training programme. To assess interobserver variation, segmentation of each CT image was performed individually by two or more observers. To assess intraobserver variation, three of the observers did repeated segmentations of the images. The distribution of variation between subjects, observers and random noise was estimated by a mixed effects model. Inter- and intraobserver correlation was assessed by intraclass correlation coefficient (ICC).

**Results:**

For all four tissue compartments, the observer variations were far lower than random noise by factors ranging from 1.6 to 3.6 and those between subjects by factors ranging from 7.3 to 186.1. All interobserver ICC was ≥ 0.938, and all intraobserver ICC was ≥ 0.996.

**Conclusions:**

Body composition segmentation showed a very low level of operator dependability. Multiple observers may interchangeably perform this task with highly reproducible results.

**Electronic supplementary material:**

The online version of this article (10.1186/s41747-019-0122-5) contains supplementary material, which is available to authorized users.

## Key points

- Body composition data may predict development and progression and aid treatment of noncommunicable diseases such as type 2 diabetes mellitus and cancer.
- Semiautomated body composition segmentation on abdominal CT images showed a very low inter- and intraobserver variation (intraclass correlation coefficient from 0.938 to 0.996).
- Semiautomated body composition segmentation may be performed by non-radiologists after a short period of training.

## Background

Computed tomography (CT) is part of the routine work-up in many patient groups. With special image segmentation software, high-precision data on body composition, i.e. the quantification and distribution of different tissues, may be extracted from these images [1].

Body composition states such as obesity and sarcopenia are associated with the risk of development and progression of noncommunicable diseases as well as overall survival [2–6]. Excess adipose tissue in the abdominal region increases the risk of type 2 diabetes mellitus (T2DM), cardiometabolic diseases and some cancers [2, 7]. Sarcopenia is a recognised diabetic and oncologic complication, and insulin resistance is a central mechanism both in sarcopenia and obesity-related diseases [8–12]. Central obesity with sarcopenia, i.e. sarcopenic obesity, may increase the effects on metabolic disorders, cardiovascular diseases and mortality [13].

Image segmentation is increasingly used as a research tool in areas such as oncology, endocrinology, cardiovascular disease, nutrition, obesity and ageing [1, 14, 15]. Semiautomated methods are faster, easier and more versatile than manual delineation and without loss of precision [14, 16–18]. Segmentation is validated against cadaver studies and offers advantages to dual-energy X-ray absorptiometry scans and bioelectric impedance analysis [19–21].

Since the cross-sectional areas at the third lumbar vertebra are linearly related to whole body mass of muscle, visceral adipose tissue (VAT) and subcutaneous adipose tissue (SAT), a single axial image is often acquired in research settings to reduce cost or radiation exposure [1, 22, 23].

Body composition analysis is also valuable in current clinical practice such as identification of cachexia in patients with cancer and is included in the newly published GLIM (Global Leadership Initiative on Malnutrition) criteria for malnutrition [24–28]. Assessment of nutritional status in patients with noncommunicable diseases such as T2DM and cancer may facilitate personalised and precision medicine and greatly impact treatment and prognosis [2, 7, 13, 25].

In order to acquire large amounts of data, for clinical or research purposes, the process of image segmentation and analysis must be quick and precise. It would be a practical advantage if image segmentation could be performed by a group of personnel interchangeably rather than one dedicated person.

Our aim was to evaluate the inter- and intraobserver variation of semiautomated body composition segmentation of CT images in both healthy and diabetic subjects, to assess whether multiple observers may interchangeably perform segmentation with comparable results.

## Methods

### Ethics and study population

Participant consent and approval from the Regional Committee for Medical and Health Research Ethics were previously obtained.

We obtained CT images of 41 subjects with T2DM enrolled in the Diabetes-study [29] and 91 healthy male subjects from the INFO-study [30]. The Diabetes-study population was aged 29 to 45 years (median 41), 49% males, with a mean body mass index (BMI) of 34.0 kg/m^2^. The INFO-study population was aged 38 to 45 years (median 40), all males, with a mean BMI of 26.4 kg/m^2^.

### Computed tomography

Anonymised, unenhanced single abdominal CT images were acquired. In the Diabetes-study, CT images were obtained with a Somatom Volume Zoom, 4-slice CT scanner (Siemens Healthineers, Erlangen, Germany) at 5 cm above L4/L5 level in women and 10 cm above L4/L5 level in men with 120 kVp, 100 mAs and slice thickness 4 mm. In the INFO-study, CT images were obtained with a Somatom Sensation 64, 64-slice scanner (Siemens Healthineers, Erlangen, Germany) at L3/L4 level with 120 kVp, 200 mAs and slice thickness 5 mm.

### Image analysis

Semiautomated body composition segmentation of the CT images was performed with the SliceOmatic software package (v 5.0 rev 7b, Tomovision, Magog, QC, Canada).

Body composition segmentation included four tissue compartments: abdominal muscle compartment (AMC), inter- and intramuscular adipose tissue (IMAT), VAT and SAT.

The segmentation was performed by four observers: three radiographers (radiology technicians) and one oncology resident. The resident had previously received training in the use of SliceOmatic at the University of Alberta hospital, Edmonton, AB, Canada. Over the course of 2 weeks, the resident held three 1-h teaching sessions for the technicians. This was followed by 7 to 12 h of practical training in the use of the software with individual feedback from the resident and two radiologists supervising the study.

**Fig. 1:**
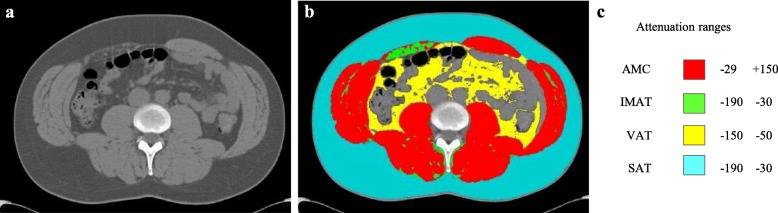
Example of segmentation. Original computed tomography image (**a**). Segmented image (**b**). Attenuation ranges defined for each segmented tissue (**c**)

Segmentation was performed according to *the Alberta protocol*, defined and used at the Alberta Hospital (AB, Canada), as shown in Fig. 1 [31]. By this definition, AMC is *muscle tissue free of adipose tissue*, not *anatomical muscle* which may include intramuscular fat, and IMAT was segmented separately as adipose tissue within the muscle fasciae. For each tissue, segmentation was restricted to the following predefined attenuation ranges: − 29 to 150 Hounsfield Units (HU) for AMC, − 190 to − 30 HU for IMAT and SAT and − 150 to − 50 HU for VAT [19, 31, 32].

**Fig. 2:**
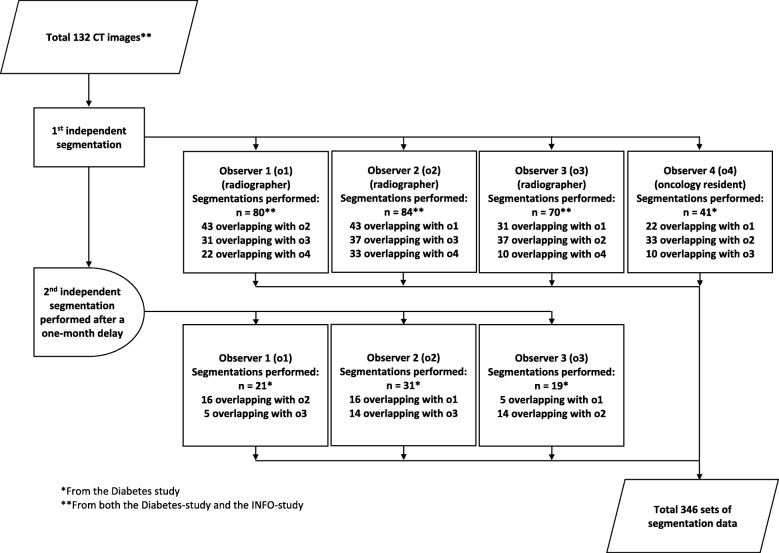
Distribution of segmentation between observers. Segmentation of each CT image was performed by two or more observers to evaluate interobserver variation. A repeated segmentation of a subset of CT images was performed after a 1-month delay to evaluate intraobserver variation

The three radiographers (observers 1, 2 and 3) performed segmentation of all the CT images from both studies. They were organised into alternating pairs so that each image was analysed independently by two radiographers. To evaluate intraobserver variation, the three radiographers performed a second segmentation of the same images from the Diabetes-study, after a 1-month delay. The oncology resident (observer 4) performed segmentation of all images from the Diabetes-study. The observers were blinded to each other’s results and their own previous results. A flow chart describing the distribution of performed segmentations between the observers is shown in Fig. 2.

From the different modes, available in the SliceOmatic software, the observers utilised the *Region growing*-mode with the *Paint* and *Grow 2D* options. This mode allowed the users to delineate the different types of tissue based on predefined attenuation ranges. The tissue compartments were tagged in a specific order, starting with AMC followed by SAT, VAT and IMAT. Although the software could delineate these four compartments semiautomatically using the *Grow 2D* option, all the segmented compartments were manually adjusted in each image with the *Paint* tool to ensure that the compartments had been segmented correctly, especially around the muscle with nearby tissues of similar density such as bowels or kidneys, but also around vertebra and IMAT.

### Exclusion criteria

CT images with inferior quality due to noise, respiratory artefacts or other movement artefacts were excluded from analysis. Images where AMC was cut from the field of view (FOV) bilaterally were also excluded. In images where the oblique or transverse abdominal muscles were cut unilaterally from the FOV, AMC was estimated by segmentation of the contralateral AMC multiplied by two. In images where SAT or VAT was cut unilaterally or bilaterally from the FOV, segmentation of the affected tissue compartment was not performed.

### Statistical analysis

Segmentation data in square centimetres per tissue compartment for each image was exported from SliceOmatic to Microsoft Excel (version 14.0, Microsoft Corporation, Redmond, WA, USA) and analysed in IBM SPSS Statistics (version 23, IBM Corporation, Armonk, NY, USA) and R (version 3.3, www.r-project.org).

Descriptive statistics are presented as median, minimum, maximum and percentiles. Normal distribution of measurement data was evaluated with Q-Q plots and Shapiro-Wilk tests.

For each of the four tissue compartments, the underlying variations in the measurement data between individual subjects, individual observers and residual variation (random noise) were analysed with mixed effects models. Different levels of variation and interactions between variations were evaluated in three different potential mixed effects models named A, B and C. All models included variation between individual subjects and random noise. Additionally, model A included general observer to observer variation, model B included systematic variation between observers in how segmentation was performed and model C included both sources of variation.

Parameters for each model were estimated by restricted maximum likelihood. Model fit for the three models was evaluated by Akaike information criterion (AIC), and results from the best fitting model presented as estimated standard deviations with 95% confidence intervals.

Mixed effects models assume normally distributed residuals, which were not present in the measurement data as seen in the Q-Q plots. The efficiency of the applied estimation routine was evaluated by simulations, and a robustness analysis was performed on data transformed to achieve normally distributed residuals.

In addition to the mixed effects model estimates, intraclass correlation coefficients (ICCs) were calculated. Two-way random effect ICC was used for overall variation in segmentation results, and single measurement ICC for intraobserver variation. Confidence intervals for the two-way ICC were based on a random effects model with percentile bootstrap confidence intervals based on 10,000 replications randomly sampling subjects and observers.

Subgroup analyses were performed for the Diabetes-study and the INFO-study in order to explore differences in variations between the subject groups.

## Results

Body composition segmentation was performed on 120 of 132 CT images. Eight were excluded from analysis due to compartments being cut from the field of view, two due to respiratory or movement artefacts and two due to image noise. From the 120 images, we acquired 346 sets of segmentation data for AMC, IMAT and VAT and 338 sets for SAT.

Q-Q plots showed that none of the segmentation data were normally distributed (all Shapiro-Wilk tests *p* < 0.010). Descriptive statistics of segmentation data of the four tissue compartments are shown in Table 1.

**Table 1:** Descriptive statistics of segmentation data per study

Compartment	*n*	Median (cm^2^)	Min-max (cm^2^)	*P*_25%_–*P*_75%_ (cm^2^)
AMC				
Total	346	170.1	65.4–250.1	141.5–190.0
Diabetes-study	186	142.7	65.4–201.2	132.1–164.4
INFO-study	160	189.6	150.1–250.1	177.4–189.6
IMAT				
Total	346	5.3	0.2–54.9	2.7–10.5
Diabetes-study	186	9.0	1.4–54.9	5.6–15.2
INFO-study	160	2.9	0.2–15.6	1.6–4.7
VAT				
Total	346	124.3	8.0–297.4	68.8–188.9
Diabetes-study	186	173.9	32.3–297.4	123.2–217.2
INFO-study	160	69.5	8.0–266.6	35.0–119.2
SAT				
Total	338	209.4	38.8–609.0	121.6–334.4
Diabetes-study	183	278.7	74.6–609.0	197.0–415.5
INFO-study	155	153.5	38.8–432.0	96.7–208.7

*P*_*25%*_ 25 percentile, *P*_*75%*_ 75 percentile, *AMC* abdominal muscle compartment, *IMAT* inter- and intramuscular adipose tissue, *VAT* visceral adipose tissue, *SAT* subcutaneous adipose tissue

For each of the four tissue compartments, AIC showed best fit for model A, modelling only general observer to observer variation (Table 2). Therefore, systematic variation between observers in how segmentation was performed was not included in the final mixed effects model.

**Table 2:** Model fit evaluated by Akaike information criterion (AIC) for three mixed effects models

Model	AMC	IMAT	VAT	SAT
A: Only general observer to observer variation	2240	1813	2594	2508
B: Only systematic variation between observers in how segmentation was performed	2247	1866	2635	2527
C: Full model (A + B)	2242	1815	2596	2510

Data are AIC values. Lower AIC values indicate better model fit

*AMC* abdominal muscle compartment, *IMAT* inter- and intramuscular adipose tissue, *VAT* visceral adipose tissue, *SAT* subcutaneous adipose tissue

**Fig. 3:**
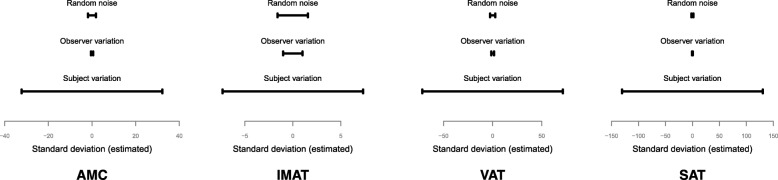
Distribution of variation between random noise, observer and subject for each segmented tissue. *AMC* abdominal muscle compartment, *IMAT* inter- and intramuscular adipose tissue, *VAT* visceral adipose tissue, *SAT* subcutaneous adipose tissue

For both studies combined, the variations between observers were consistently less than variation between subjects for all four tissue compartments by a factor of 7.3 to 186.1. Variations between observers were also less than random noise by a factor of 1.6 to 3.6 (Table 3, Fig. 3). Mixed effects model analysis yielded results with very similar interpretations on non-transformed data (Table 3) and data transformed to achieve normally distributed residuals (Additional file 1: Table S1). Simulations of non-normal distributed data showed efficient restricted maximum likelihood estimates also with similar violations of the normally distribution assumption as seen in our data (results not presented).

**Table 3:** Distribution of variation between subjects, observers and random noise per study

Compartment	Subjects		Observers		Random noise	
	SD	95% CI	SD	95% CI	SD	95% CI
AMC						
Total	32.2	28.5–36.5	0.5	0.2–1.6	1.8	1.7–2.0
Diabetes-study	29.3	23.6–36.5	0.4	0.0–1.3	1.6	1.4–1.8
INFO-study	20.2	17.4–23.6	0.6	0.0–2.7	2.2	1.9–2.7
IMAT						
Total	7.3	6.5–8.3	1.0	0.5–2.5	1.6	1.4–1.7
Diabetes-study	10.0	8.0–12.5	1.0	0.5–2.8	1.7	1.6–2.0
INFO-study	2.5	2.1–3.0	0.8	0.3–2.0	1.2	1.0–1.4
VAT						
Total	71.6	63.3–81.2	1.4	0.7–4.2	2.6	2.4–2.9
Diabetes-study	59.1	47.7–73.8	1.2	0.5–3.8	2.8	2.5–3.1
INFO-study	60.1	51.7–70.1	1.8	0.8–7.0	2.2	1.9–2.6
SAT						
Total	130.3	114.8–148.2	0.7	0.3–2.0	1.9	1.7–2.1
Diabetes-study	148.5	119.2–186.3	0.7	0.3–2.1	1.8	1.6–2.1
INFO-study	89.7	76.8–105.1	0.9	0.3–3.8	1.9	1.7–2.3

*SD* standard deviation, *CI* confidence interval, *AMC* abdominal muscle compartment, *IMAT* inter- and intramuscular adipose tissue, *VAT* visceral adipose tissue, *SAT* subcutaneous adipose tissue

For both studies combined, the interobserver ICC ranged from 0.938 to 1.000 for all four compartments, with IMAT scoring the lowest (Table 4). All intraobserver ICC ranged from 0.996 to 1.000.

**Table 4:** Inter- and intraobserver variation measurements

Compartment	Intraobserver ICC^a^	Interobserver ICC (95% CI)
AMC		
Total	0.997–0.999	0.997 (0.995–0.999)
Diabetes-study		0.997 (0.994–0.999)
INFO-study^a^		0.987 (0.978–0.999)
IMAT		
Total	0.997–0.999	0.938 (0.879–0.992)
Diabetes-study		0.961 (0.890–0.994)
INFO-study^a^		0.759 (0.568–1.000)
VAT		
Total	0.998–1.000	0.998 (0.998–1.000)
Diabetes-study		0.997 (0.995–1.000)
INFO-study^a^		0.998 (0.996–1.000)
SAT		
Total	1.000–1.000	1.000 (1.000–1.000)
Diabetes-study		1.000 (1.000–1.000)
INFO-study^a^		0.999 (0.999–1.000)

*ICC* intraclass correlation coefficient, *CI* confidence interval, *AMC* abdominal muscle compartment, *IMAT* inter- and intramuscular adipose tissue, *VAT* visceral adipose tissue, *SAT* subcutaneous adipose tissue

^a^Observers 1, 2 and 3

### Subgroup analysis

For the Diabetes-study, the variations between observers were consistently less than the variations between subjects for all four tissue compartments by a factor of 10.0 to 212.1 (Table 3). The variations between observers were also less than random noise by a factor of 1.8 to 4.0. For all four compartments, the interobserver ICC ranged from 0.961 to 1.00 (Table 4).

For the INFO-study, the variations between observers were consistently less than the variations between subjects for all four tissue compartments by a factor of 3.1 to 99.7. The variations between observers were also less than random noise by a factor of 1.2 to 3.7. For IMAT, the interobserver ICC was 0.759, and for the remaining three compartments ICC ranged from 0.987 to 1.00.

## Discussion

Our results show that after a short period of training non-radiologist physicians and radiographers can perform semiautomated segmentation of body composition on abdominal CT images with close to identical results.

Van Vugt et al. [17] showed similar results with close to perfect ICCs for inter- and intraobserver agreement. However, their observers had extensive experience in skeletal muscle and adipose tissue area measurement, whereas our inexperienced technicians produced similar results with only a short period of training.

Our results underline that the intuitive nature of the software and the standardised process of semiautomated segmentation produces consistent results even when performed by operators with less radiological experience. This facilitates segmentation of larger number of images without loss of data quality, which increases the value of this method, whether used for patient follow-up in clinical settings or measurements of research endpoints.

From the three mixed effects models, AIC consistently showed superior fit for model A, with only general observer variation. This strengthens the assumption that there was a variation between observers, but no systematic variation between observers in how segmentation was performed.

Both the mixed effects model and the ICC showed, consistently for all four tissue compartments, that observer variation was less than variation due to random noise and negligible compared to the variation between individual subjects. Consequently, a single dedicated person is not necessary for the acquisition of reliable segmentation data, and segmentation may be performed interchangeably by any member of a group of trained radiographers. This allows for greater flexibility and makes feasible the acquisition of greater amounts of data or even the future adoption into clinical practice.

In our experience, segmentation of AMC and SAT was relatively straightforward, which is supported by our data. The slightly higher observer variation observed for VAT in the mixed effects model may indicate that segmentation of this compartment is more demanding than that of AMC and SAT, which is in line with our experience. Structures in the same anatomical space as VAT, such as the viscera, the mesentery, the intestinal wall and fatty contents of the intestines, may complicate segmentation.

The slightly lower interobserver ICC for IMAT may primarily be due to the relatively small variation in this tissue compartment between subjects compared to AMC, VAT and SAT. Contributing factors may be the relatively small area of IMAT in each image and a less stringent definition of this compartment. These explanations are further supported by the subgroup analyses specifically showing a lower interobserver ICC for IMAT in the INFO-study subject group, although with a wide confidence interval. The median area of IMAT in the INFO-study was approx. one third compared to the Diabetes-study. Hence, the relative effect of observer variation was larger in the IMAT measurement resulting in a lower ICC. However, in our opinion, the subgroup analyses confirm that our results are valid for both groups separately.

We decided to present the results of the mixed effects model even though the requirement of normally distributed residuals was not met. Maximum likelihood estimation on medium-sized datasets tends to give reasonable estimates even with some violations of the modelling assumptions, which we confirmed with simulations. The analysis of non-transformed data allowed us to present the results as standard deviations, which could be compared across the segmented compartments and are easier to understand and interpret.

In order to control the robustness of the method, we carried out a sensitivity analysis with the same model on transformed data with approximately normally distributed residuals. This sensitivity analysis showed similar results, confirming the validity of our results, though in a format which is more difficult to understand and interpret.

Limitations of our study should be taken into account. Our study was limited to one, specific software, and our results may not apply perfectly to other segmentation tools. Furthermore, we did not account for variation associated with image acquisition such as the level or angle of the CT slice [1]. In addition, the exclusion of 12 images due to artefacts and noise show that not all acquired images are suitable for segmentation and that standardised acquisition protocols are necessary. Furthermore, the time spent performing segmentation was not specifically measured.

We conclude that semiautomated body composition segmentation using SliceOmatic showed a very low level of operator dependability. Hence, multiple observers may interchangeably perform body composition segmentation of abdominal CT with close to identical results in a clinical or research setting.

## Additional file

Additional file 1: **Table S1.** Distribution of variation between subjects, observers, and random noise for transformed, normally distributed data. (DOCX 18 kb)

## Data Availability

The datasets used and analysed during the current study are available from the corresponding author on reasonable request.

## Abbreviations

AIC: Akaike information criterion
AMC: Abdominal muscle compartment
BMI: Body mass index
CT: Computed tomography
ICC: Intraclass correlation coefficient
IMAT: Inter- and intramuscular adipose tissue
SAT: Subcutaneous adipose tissue
T2DM: Type 2 diabetes mellitus
VAT: Visceral adipose tissue
