# Supplementary material for: Reproducibility of semiautomated body composition segmentation of abdominal computed tomography: a multiobserver study
Source: Eur Radiol Exp. 2019 Oct 30;3:42. doi: 10.1186/s41747-019-0122-5 (PMC6820626; doi:10.1186/s41747-019-0122-5)
Supplement: Supplementary file 1 — Table S1. Distribution of variation between subjects, observers, and random noise for transformed, normally distributed data. (DOCX 18 kb) [file 41747_2019_122_MOESM1_ESM.docx]

**Additional file 1: Table S1.** Distribution of variation between subjects, observers, and random noise for transformed, normally distributed data.

| Compartment | Equation for transformation^*^ | Subjects^†^ | Observers^†^ | Random noise^†^ |
| --- | --- | --- | --- | --- |
| AMC | $x^{1.5}$ | 621.10 | 9.21 | 36.51 |
| IMAT | $\log(x)$ | 0.92 | 0.13 | 0.23 |
| VAT | $sgn\left[ x-mean\left( x \right) \right]*{[x-mean\left( x \right)]}^{1.5}$ | 706.17 | 15.18 | 30.18 |
| SAT | $sgn[x^{0.5}-mean(x^{0.5})*{[x^{0.5}-mean\left( x^{0.5} \right)]}^{1.25}$ | 6.65 | 0.04 | 0.09 |

**^*^**”*sgn*” is the sign of the given equation. “*mean (x)*” is the mean of the x. **^†^**Numbers are standard deviations

*AMC* Abdominal muscle compartment, *IMAT* Inter- and intramuscular adipose tissue, *VAT* Visceral adipose tissue, *SAT* Subcutaneous adipose tissue.
